# Diversity of Soil Fungi, Entomopathogenic Fungi and Their Driving Factors in the Eastern Tanggula Mountains in the Qinghai–Tibet Plateau

**DOI:** 10.3390/microorganisms14071507

**Published:** 2026-07-10

**Authors:** Langba Danzeng, Gadan Langjie, Yao Xiao, Danzeng Luobu, Jinxing Li, Dun Wang

**Affiliations:** Key Laboratory of Plant Protection Resources and Pest Management of Ministry of Education, College of Plant Protection, Northwest A&F University, Yangling 712100, China; langbadanzeng@foxmail.com (L.D.);

**Keywords:** alpine grassland, entomopathogenic fungi, fungal community structure, elevational gradient, high-throughput sequencing

## Abstract

This study aims to elucidate the soil fungal community structure, the distribution of entomopathogenic fungi (EPF) resources, and their driving mechanisms in the alpine grassland of the northern side of the eastern Tanggula Mountains in the Qinghai–Tibet Plateau. Five representative sites were established along an elevational gradient of 4000–5100 m, and 15 soil samples were collected. High-throughput sequencing (ITS region) and an insect baiting method were combined to analyze fungal community composition and diversity. LEfSe analysis, random forest modeling, redundancy analysis (RDA), and network analysis were employed to reveal the environmental driving mechanisms of community assembly. A total of 412 fungal species belonging to 303 genera were annotated, with Ascomycota as the dominant phylum. At the high-elevation site Jie-duo Town, Zaduo County (ZD-JD), the cold-tolerant ectomycorrhizal fungus *Inocybe* was absolutely dominant (17.18%). The low-elevation site Dong-ba Town, Nangqian County (NQ-DB), was enriched with the saprotrophic genus *Mortierella* (10.34%) and EPF taxa. The mid-elevation site Zhuo-xiao Town, Nangqian County (NQ-ZX), was characterized by *Solicocozyma* (11.88%) and exhibited the highest network complexity. Alpha diversity decreased with increasing elevation. A total of 35 EPF strains were isolated via the baiting method, predominantly *Metarhizium anisopliae*, *M. brunneum*, and *M. robertsii*, with the highest number of isolates (16 strains) recovered from NQ-DB. Elevation indirectly drove community assembly by regulating temperature and plant productivity. The soil fungal community structure in the alpine grassland of the eastern Tanggula Mountains exhibits significant spatial heterogeneity along the elevational gradient. Low-elevation habitats serve as hotspots for EPF resources, while high-elevation isolates harbor potential stress-resistance genes. The combination of culture-dependent and sequencing-based approaches provides a reliable strategy for microbial resource surveys in extreme environments.

## 1. Introduction

As a cornerstone of subterranean ecosystems, soil microorganisms encompass a diverse array of taxa—including bacteria, fungi, archaea, and protists—serving as the primary nexus connecting soil, vegetation, and the broader environment [1,2,3]. Among these, soil fungi play a dual role: they not only drive fundamental ecological processes such as organic matter decomposition, nutrient cycling, and the enhancement of plant stress resistance, but also regulate soil carbon, nitrogen, and phosphorus biogeochemical cycling, soil organic matter retention, and complex plant–soil interaction networks. Through these mechanisms, soil fungi exert a profound influence on the stability and ecosystem service functions of terrestrial environments [4,5,6,7].

Alpine steppes are widely distributed across the high-altitude regions of the Qinghai-Xizang Plateau. These habitats are characterized by a suite of extreme environmental constraints, including perennial low temperatures, low partial pressure of oxygen, intense ultraviolet (UV) radiation, shallow and nutrient-poor soils, fluctuating hydrothermal conditions, and exceptionally brief plant growing seasons. The rigorous environmental filtering associated with these conditions has significantly reshaped microbial community structures, taxonomic compositions, and physiological metabolic strategies. Consequently, these processes have fostered unique microbial assemblages that are distinct from those found in low-altitude temperate grasslands [4,5,6,8].

Against this backdrop, the assembly of soil fungal communities in alpine steppes has been confirmed to be synergistically driven by environmental filtering and biological interactions. Regarding environmental filtering, variations in altitudinal gradients select for adaptive tolerant species, leading to reduced fungal diversity and the concentration of dominant taxa in high-altitude regions; meanwhile, seasonal precipitation fluctuations and faunal disturbances further reshape community structure and network stability [9,10]. Regarding biological interactions, plant roots can induce microbial memory in rhizosphere fungi to cope with drought [7,11,12,13], whereas entomopathogenic fungi have evolved proactive attractant strategies, actively luring hosts through the secretion of effectors and the release of volatile organic compounds such as longifolene [14,15,16]. However, existing research has largely focused on isolated factors, leaving a gap in the systematic analysis of the multidimensional coupling mechanism of plant–soil–microorganism systems. The molecular mechanisms by which fungi in high-altitude ecotones achieve a growth–defense tradeoff through genomic specializations—such as cold-shock proteins—remain to be elucidated through advanced multi-omics technologies [17,18,19,20].

Of significant interest is the role of entomopathogenic fungi (EPF) as a core resource for green biocontrol. Recent years have witnessed a series of breakthroughs in the study of their pathogenic mechanisms: *Beauveria bassiana* var. grandispora has been shown to tolerate the highly alkaline environment of the midgut via the foregut pathway to successfully infect its host [21]; the virulence factor Fkp1 works synergistically with cross-kingdom microRNAs (milR1) to suppress insect immune recognition and the Toll signaling pathway [16]; and the formation of appressoria can induce metabolic remodeling and programmed cell death in the host [22]. Collectively, these findings construct a comprehensive pathogenic model encompassing “attraction-multipathway invasion-immune evasion-metabolic reprogramming,” establishing a robust theoretical foundation for the development of engineered fungal strains. However, systematic reports are still lacking regarding whether the extreme alpine environment of the Qinghai–Tibet Plateau harbors EPF germplasm resources characterized by low-temperature tolerance and high ultraviolet (UV) resistance.

Despite continuous breakthroughs in understanding the pathogenic mechanisms of entomopathogenic fungi (EPF), the distribution patterns of soil fungal communities along elevational gradients in alpine steppe remain poorly understood, and systematic surveys across a continuous elevational gradient on the northern flank of the eastern Tanggula Mountains are particularly lacking. To address these research gaps, this study focused on the 4000–5000 m elevational belt on the northern side of the eastern Tanggula Mountains. By integrating high-throughput sequencing and culturomics, we aim to characterize the diversity and network features of soil fungal communities, evaluate the abundance of EPF resources, and elucidate their environmental driving mechanisms in this region. This work is expected to provide a scientific basis for the development of green pest control resources in alpine steppe and for research on ecosystem responses to climate change.

## 2. Materials and Methods

### 2.1. Study Area Overview and Sampling

The study area is situated at the northern foothills of the eastern Tanggula Mountains in the hinterland of the Qinghai-Xizang Plateau, encompassing five representative sampling sites across Zadoi County, Nangqen County, and Yushu City within the Yushu Tibetan Autonomous Prefecture, Qinghai Province (Table 1). The region is characterized by a plateau cold-temperate semi-humid climate, with an average annual temperature ranging from −3 to 3 °C and annual precipitation between 250 and 450 mm. The predominant soil type is alpine meadow soil (Cryumbrept).

Sampling was conducted from late June to early July 2024. At each site, three independent replicates were established (with a minimum inter-plot distance of >50 m). Soil samples were collected from the 5–20 cm depth layer using the “five-point sampling method” (each composite sample was composed with five sub-samples collected from five points with the sub-plot.). The collected soil was sieved through a 10-mesh screen and homogenized to yield a total of 15 composite soil samples, which were subsequently transported to the laboratory via a cold chain maintained at −10 °C.

### 2.2. Experimental Methods

#### 2.2.1. High-Throughput Sequencing and Bioinformatics Analysis

DNA extraction and sequencing were commissioned to a commercial service company (Biomarker Technologies, Beijing, China). The fungal ITS region was amplified via PCR using the primer pair ITS1F (5′-CTTGGTCATTTAGAGGAAGTAA-3′) and ITS2R (5′-TGTGTTCTTCATCGATG-3′), followed by paired-end sequencing on the Illumina MiSeq platform. Raw sequences underwent rigorous quality control before being clustered into Operational Taxonomic Units (OTUs) at a 97% similarity threshold using QIIME2 (Version 2024.2). Taxonomic annotation was performed based on the UNITE database.

#### 2.2.2. Isolation and Identification of Entomopathogenic Fungi

Entomopathogenic fungi were isolated using the insect-bait method with *Tenebrio molitor* larvae. Approximately 150 g of soil was transferred into sterile tissue culture flasks, into which 10 healthy larvae were introduced and incubated at 15–20 °C. Upon cadaver mummification, the larvae were surface-sterilized and inoculated onto SDAY medium (Sabouraud Dextrose Agar Yeast Extract: Peptone 10.0 g, Yeast Extract 10.0 g, Dextrose 40.0 g and Agar 20.0 g in 1.0 L distilled water) supplemented with antibiotics. Pure cultures were obtained through successive subculturing of single colonies. Strain identification was conducted through morphological observation coupled with molecular analysis of the by two gene fragments of *ITS* and *LSU*. The PCR primers for *ITS* region were ITS1F (5′-TCCGTAGGTGAACCTGCGG-3′) and ITS4R (5′-TCCTCCGCTTATTGATATGC-3′). The PCR primers for *LSU* region were LSU5F (5′-ATCCTGAGGGAAACTTC-3′) and LSU0R (5′-GTACCCGCTGAACTTAAGC-3′). The specific procedure for morphological and molecular identification of culturable fungi refer to Meng et al. [23].

#### 2.2.3. Data Processing and Statistical Analysis

Statistical analyses were performed using R language (version R 4.4.2; vegan, phyloseq, randomForest packages). Alpha diversity indices (Shannon, Chao1, etc.) were calculated. PLS-DA was used for Beta diversity analysis. LEfSe analysis was employed to identify biomarker taxa. Random forest modeling assessed genus-level importance. Redundancy analysis (RDA) and network analysis were used to parse environmental driving mechanisms.

## 3. Results

### 3.1. Soil Entomopathogenic Fungal Community Structure Characteristics

High-throughput sequencing yielded a total of 412 annotatable fungal species, belonging to 303 genera, 169 families, and 86 orders, underscoring the exceptionally high fungal taxonomic diversity within the study area. At the phylum level, Ascomycota exerted absolute dominance across all sampling sites, followed by Basidiomycota and Zygomycota. Notably, entomopathogenic-related taxa, such as the genera *Metarhizium* and *Beauveria*, were primarily nested within the order *Hypocreales*.

**Figure 1:**
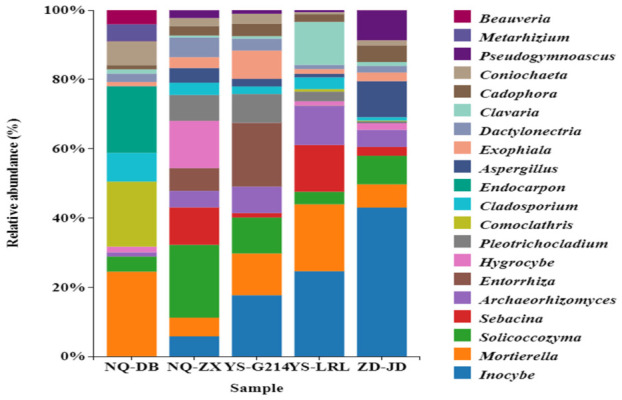
Bar plot of relative abundance at the genus level.

The distribution of dominant taxa at the genus level (Figure 1) exhibited pronounced spatial heterogeneity across the different sampling sites:

ZD-JD (High Altitude): The genus *Inocybe* emerged as the absolute dominant group, with a relative abundance as high as 17.18%, demonstrating a robust physiological adaptation to extreme alpine environments.

NQ-DB (Low Altitude): *Mortierella* exhibited the highest abundance (10.34%). Crucially, *Beauveria* (1.73%) and *Metarhizium* (2.02%) were significantly enriched in this locality.

NQ-ZX (Mid-Altitude): *Solicocozyma* constituted the most dominant taxon (11.88%), resulting in a distinct community structure.

YS-LRL and YS-G214: These sites displayed a relatively balanced community architecture without the absolute monopoly of a single genus; *Inocybe* and *Mortierella* were found to be more uniformly distributed here.

Above results showed that the relative abundances of these dominant genera varied markedly among sampling sites, exhibiting pronounced spatial heterogeneity in their distribution.

### 3.2. Diversity Analysis

#### 3.2.1. Alpha Diversity

Alpha diversity indices reflected the complexity of local communities at each site (Table 2). Yushu Liurilong (YS-LRL) had the highest Shannon index (3.26) and Evenness index (0.81), indicating minimal habitat disturbance and optimal species coexistence. Nangqian Dongba (NQ-DB) had the highest Chao1 index (143), predicting the greatest potential species richness and identifying it as a hotspot for fungal resources. Zaduo Jieduo (ZD-JD) had the lowest indicators across the board, confirming that the high-altitude environment exerted significant filtering pressure on microbial diversity, leading to simplified community structure. The rarefaction curves of all collected soil samples gradually approached a plateau, indicating that the sampling effort was sufficient and the sequencing depth was reasonable.

#### 3.2.2. Beta Diversity

**Figure 2:**
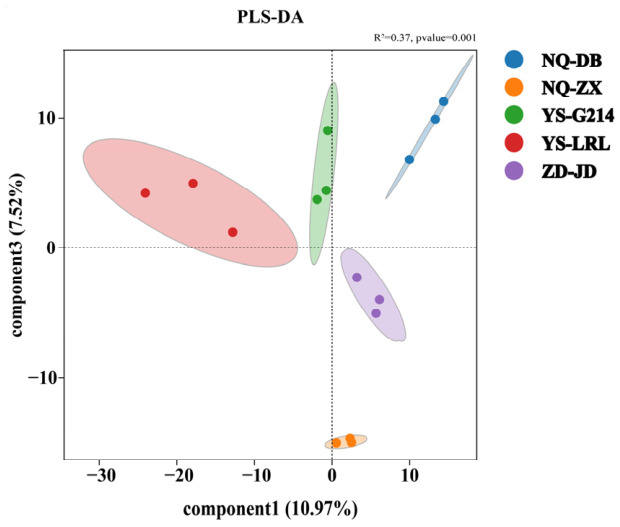
PLS-DA analysis.

PLS-DA analysis revealed overall divergence in community structure among sites (Figure 2). YS-LRL and YS-G214 clustered closely, indicating highly similar fungal community structures driven by comparable microclimates and vegetation. NQ-ZX exhibited distinct marginal clustering in the two-dimensional space, differing most significantly from other sites, suggesting that the mid-altitude ecotone formed a unique niche. ZD-JD and NQ-DB differed significantly, confirming that elevation gradient is the primary geographic barrier causing microbial community differentiation. The PLS-DA plot revealed substantial differences in fungal community composition among soil samples from different locations. Notably, YS-G214, YS-LRL, and ZD-JD exhibited relatively similar compositional profiles.

### 3.3. Identification of Entomopathogenic Fungi from Soil Samples

Using the *Tenebrio molitor* baiting method, a total of 35 entomopathogenic fungal strains were isolated and identified in this study (Table 3). The genus *Metarhizium* emerged as the absolute dominant group, accounting for 33 of the isolates. This group comprised 14 strains of *M. anisopliae*, 11 strains of *M. brunneum*, and 6 strains of *M. robertsii*. Other identified taxa included a single strain of *Pleurocordyceps sinensis* and one strain of *Mortierella alpina*.

The distribution of isolates revealed that Site NQ-DB harbored the largest number of strains (16 in total), suggesting that the relatively warm and moist low-elevation environment favors the establishment and reproduction of entomopathogenic fungi.

### 3.4. Ecological Driving Mechanisms of Entomopathogenic Fungi

#### 3.4.1. Biomarker Taxa Identification (LEfSe Analysis)

**Figure 3:**
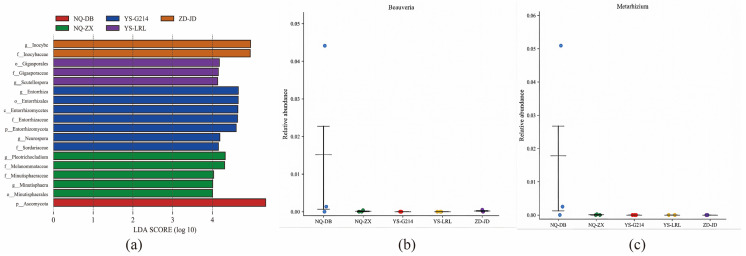
LEfSe analysis of differential fungal taxa among sampling sites. (**a**) LDA score; (**b**) *Beauveria*; (**c**) *Metarhizium*. ZD-JD: *Inocybe* was the significant biomarker, indicating the high-altitude habitat. NQ-DB: Ascomycota and insecticidal functional genera (*Beauveria*, *Metarhizium*) were significantly enriched. NQ-ZX: *Solicocozyma* was the specific indicator genus.

LEfSe analysis (LDA > 4) further confirmed the keystone taxa of each site (Figure 3). LEfSe analysis revealed that distinct biomarker taxa were identified across different soil samples at various taxonomic levels (LDA > 4). For instance, *Inocybe* was identified as the characteristic genus in soil samples from ZD-JD, whereas *Ascomycota* was the predominant biomarker phylum in samples from NQ-DB. Moreover, comparison of the relative abundances of *Beauveria* and *Metarhizium* among different groups indicated that soil samples from NQ-DB harbored abundant resources of both entomopathogenic fungal genera.

#### 3.4.2. Random Forest and Network Analysis

**Figure 4:**
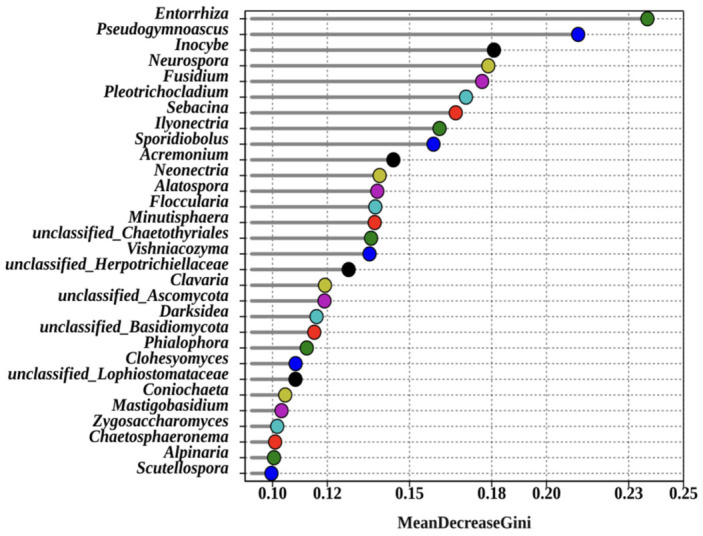
Random Forest analysis.

Random Forest modeling identified the genera *Entorrhiza*, *Pseudogymnoascus*, and *Inocybe* as the key predictor variables contributing most significantly to the differentiation between study plots (Figure 4). Correlation network analysis further revealed distinct variations in community stability across the sites (Table 4): Community Interaction and Stability: NQ-ZX exhibited the highest average degree (4.08) and clustering coefficient (0.3121), indicating that the fungal taxa in this plot possess the most robust interspecific interactions and the highest ecosystem stability. Network Connectivity: in contrast, NQ-DB displayed the lowest network density. This suggests that despite its high species richness, the community is characterized by weaker interspecific competition or relatively simplistic collaborative relationships.

Random Forest analysis at the genus level revealed that *Entorrhiza* contributed most significantly to the differentiation among samples, followed by *Pseudogymnoascus*, *Inocybe*, and others. These results indicate that the identified fungal taxa exhibit distinct geographical distribution patterns.

## 4. Discussion

The observed trend of decreasing fungal diversity with increasing elevation (lowest at ZD-JD) aligns with the High-elevation Filtering Hypothesis [24]. *Inocybe* is a typical ectomycorrhizal fungus (ECM Fungi) that forms obligate symbiotic relationships with alpine plant roots, obtaining photosynthetic carbon sources to sustain survival [25,26]. This genus exhibits significant cold tolerance and is widely distributed in cold-temperate forests and high-altitude grasslands, adapting to extreme environments such as low temperatures and intense radiation. Recent molecular phylogenetics has revealed deep evolutionary lineages and cryptic diversity within this genus, yet ecological function research remains focused on its key role in nutrient exchange and community assembly as a rhizosphere symbiotic partner. Its strict dependence on plant roots and preference for alpine habitats make it an ideal indicator taxon for analyzing subterranean ecological processes in high-altitude regions [10,27,28,29]. Conversely, the high fungal abundance at NQ-DB may be attributed to favorable low-altitude hydrothermal conditions, high plant productivity, and increased litter input, supporting more saprophytic fungi like *Mortierella*.

High-throughput sequencing revealed a low relative abundance of EPF (<2%), yet the baiting method successfully isolated 35 strains, corroborating the complementarity of traditional cultivation and HTS in fungal resource exploration. The low-altitude site NQ-DB exhibited the highest species richness, with abundance significantly higher than other sites, and yielded 16 entomopathogenic fungal isolates via baiting, indicating that warm, humid, and vegetated low-altitude habitats favor EPF colonization and enrichment [16,30]. Specifically, the suitable temperature range (15–25 °C) at NQ-DB provided an ideal microenvironment for *Metarhizium* spore germination and infection. Elevation indirectly drives fungal community assembly by regulating temperature and plant productivity, acting as a dominant ecological factor rather than a sole determinant of EPF distribution. Notably, *M. robertsii* and *M. anisopliae* isolated in this study are globally recognized important biocontrol agents [31,32]. Their high-altitude origin implies these strains may carry special stress-resistance genes, e.g., cold tolerance and UV resistance, holding potential value for further exploitation.

Furthermore, the significant enrichment of *Solicocozyma* (11.88%) at the mid-altitude site NQ-ZX (4452 m) is closely related to niche differentiation at the ecotone transitioning from subalpine meadow to alpine grassland. The high habitat heterogeneity of the ecotone provides unique competitive advantages for yeast-like fungi [33]. Network analysis further substantiates this: NQ-ZX exhibited the highest average degree (4.08) and clustering coefficient (0.3121), indicating tight interspecific interactions and strong network stability, reflecting the ecological strategy of transitional zone communities adapting to variable environments through enhanced interspecies cooperation [34,35]. In contrast, although NQ-DB had the highest species richness, its network density was the lowest (0.0360) with a loose structure, implying that abundant resources may weaken interspecific interaction intensity [36]. Thus, low altitudes support high species coexistence, while mid-altitude ecotones construct more resilient ecosystems under resource constraints.

## 5. Conclusions

This study systematically analyzed the soil fungal community structure and distribution patterns of entomopathogenic fungi (EPF) resources along an elevational gradient of 4000–5100 m in the alpine grassland at the northern foot of the eastern Tanggula Mountains, utilizing a combination of high-throughput sequencing and culturomics. A total of 412 fungal species belonging to 303 genera were annotated, with Ascomycota as the absolutely dominant phylum. Community composition exhibited significant spatial heterogeneity: the high-elevation site ZD-JD was dominated by the cold-tolerant ectomycorrhizal fungus *Inocybe* (17.18%); the low-elevation site NQ-DB was enriched with the saprotrophic genus *Mortierella* and EPF taxa; and the mid-elevation site NQ-ZX was characterized by *Solicocozyma* (11.88%) and possessed the highest network complexity. Elevation served as the dominant factor driving community differentiation, indirectly shaping diversity patterns by regulating temperature and plant productivity. NQ-DB acted as a hotspot for EPF resources, yielding 16 of the 35 strains isolated via baiting, covering globally important biocontrol species such as *M. anisopliae*, *M. brunneum*, and *M. robertsii*, confirming that low-altitude hydrothermal habitats are most conducive to EPF colonization. Although sequencing indicated EPF abundance was <2%, the cultivation method effectively captured low-abundance functional groups, highlighting the complementarity of both approaches. This study fills the gap in EPF resource surveys in the region and constructs a multidimensional analytical framework of “altitude-environment-community-resources”. Future research should focus on genomic analysis and functional verification of stress resistance in isolated strains, expand temporal monitoring and multi-trophic level interaction studies, and promote the development of localized biocontrol agents for alpine regions.

## Figures and Tables

**Table 1:** Basic information of sampling sites.

Site ID	Location	Coordinates	Elevation (m)
ZD-JD	Jie-duo Town, Zaduo County	93°38′ E, 33°14′ N	5042
NQ-DB	Dong-ba Town, Nangqian County	96°38′ E, 32°21′ N	4001
NQ-ZX	Zhuo-xiao Town, Nangqian County	97°28′ E, 32°01′ N	4452
YS-LRL	Liu-ri-long, Shanglaxiu Town, Yushu City	96°97′ E, 33°03′ N	4225
YS-G214	G214, Garila mountain pass, Yushu City	97°20′ E, 33°40′ N	4333

**Table 2:** Alpha diversity indices of fungal communities across sampling sites.

Site ID	Shannon Index	Chao1 Index	Evenness Index
ZD-JD	2.38	110	0.61
NQ-DB	3.12	143	0.78
NQ-ZX	2.51	102	0.65
YS-LRL	3.26	135	0.81
YS-G214	3.04	127	0.79

**Table 3:** Entomopathogenic fungal strains isolated via insect bait method.

Fungal Species	Number of Isolates	Main Sampling Sites of Distribution
*Metarhizium anisopliae*	14	ZD-JD, NQ-DB, YS-LRL
*Metarhizium brunneum*	11	NQ-DB, YS-LRL, YS-G214
*Metarhizium robertsii*	6	ZD-JD, NQ-DB
*Pleurocordyceps sinensis*	1	NQ-DB
*Mortierella alpina*	1	NQ-DB
*Fusarium verticillioides*	2	NQ-DB

**Table 4:** Topological parameters of fungal community correlation networks.

Site ID	Average Degree	Clustering Coefficient	Graph Density
NQ-DB	2.67	0.0702	0.0360
NQ-ZX	4.08	0.3121	0.0850
YS-G214	3.03	0.1029	0.0466
YS-LRL	3.23	0.1641	0.0529
ZD-JD	3.17	0.1521	0.0512

## Data Availability

Data is unavailable due to privacy of regional restriction.

## References

[1] Duan S., Feng G., Limpens E., Bonfante P., Xie X., Zhang L. (2024). Cross-kingdom nutrient exchange in the plant–arbuscular mycorrhizal fungus–bacterium continuum. Nat. Rev. Microbiol..

[2] Philippot L., Chenu C., Kappler A., Rillig M.C., Fierer N. (2024). The interplay between microbial communities and soil properties. Nat. Rev. Microbiol..

[3] Zhang Y., Jing M., Lyu L., Nie L., Xu X., Sun R., Xu X., Chen S., He S., Zhang Y. (2026). Principles for rigorous design and application of synthetic microbial communities. Adv. Sci..

[4] Chen H., Ju P., Zhu Q., Xu X., Wu N., Gao Y., Feng X., Tian J., Niu S., Zhang Y. (2022). Carbon and nitrogen cycling on the Qinghai–Tibetan Plateau. Nat. Rev. Earth Environ..

[5] Ren L., Huo J., Xiang X., Pan Y., Li Y., Wang Y., Meng D., Yu C., Chen Y., Xu Z. (2023). Environmental conditions are the dominant factor influencing stability of terrestrial ecosystems on the Tibetan plateau. Commun. Earth Environ..

[6] Zhao Y., Qin F., Cui Q., Li Q., Cui Y., Birks H.J.B., Liang C., Zhao W., Li H., Ren W. (2025). Three-and-a-half million years of Tibetan Plateau vegetation dynamics in response to climate change. Nat. Ecol. Evol..

[7] Wang Z., Li Z., Zhang Y., Liao J., Guan K., Zhai J., Meng P., Tang X., Dong T., Song Y. (2024). Root hair developmental regulators orchestrate drought triggered microbiome changes and the interaction with beneficial Rhizobiaceae. Nat. Commun..

[8] Wang Y., Lue Y., Lü D., Yin L., Wang X. (2024). Climate change and its ecological risks are spatially heterogeneous in high-altitude region: The case of Qinghai-Tibet plateau. Catena.

[9] Yu Y., Ru J., Lei B., Han S., Wan S., Zheng J. (2024). Distinct response patterns of soil micro-eukaryotic communities to early-season and late-season precipitation in a semiarid grassland. Soil Biol. Biochem..

[10] Zhang X., Tang Z., Yang J., Herath S., Wang Z., Wang Y., Chen G., Yue L. (2025). Plateau zokor disturbances transform the stability and functional characteristics of soil fungal communities. Geoderma.

[11] Chen P., Yu Q., Wang C., Montoya L., West P.T., Xu L., Varoquaux N., Cole B., Hixson K.K., Kim Y.m. (2025). Holo-omics disentangle drought response and biotic interactions among plant, endophyte and pathogen. New Phytol..

[12] Chen S., Wang Y., Chen B., Hou X., Liu S., He S., Qi J., Peng Z., Pan H., Liang C. (2026). Diversity triggered 2-naphthoic acid exudation recruits keystone microbial taxa to promote soybean drought tolerance. Cell Host Microbe.

[13] Qi H., Wen X., Wang Z., Yin S. (2026). Microbial memory of drought reshapes root-associated communities to enhance plant resilience. Plant Cell Environ..

[14] Crandall L., Zaman R., Duthie-Holt M., Jarvis W., Erbilgin N. (2025). Navigating the semiochemical landscape: Attraction of subcortical beetle communities to bark beetle pheromones, fungal and host tree volatiles. Insects.

[15] Tang D., Chen J., Zhang Y., Tang X., Wang X., Yu C., Cheng X., Zhang J., Shi W., Zhen Q. (2025). Engineered *Metarhizium* fungi produce longifolene to attract and kill mosquitoes. Nat. Microbiol..

[16] Tang G., Song S., Shang J., Luo Y., Li S., Wei D., Wang C. (2025). Fungal evasion of *Drosophila* immunity involves blocking the cathepsin-mediated cleavage maturation of the danger-sensing protease. Proc. Natl. Acad. Sci. USA.

[17] Chen C., Buscaill P., Sanguankiattichai N., Huang J., Kaschani F., Kaiser M., van der Hoorn R.A. (2024). Extracellular plant subtilases dampen cold-shock peptide elicitor levels. Nat. Plants.

[18] Ngou B.P.M., Wyler M., Schmid M.W., Suzuki T., Albert M., Dohmae N., Kadota Y., Shirasu K. (2025). Systematic discovery and engineering of synthetic immune receptors in plants. Science.

[19] Pushkareva E., Hejduková E., Elster J., Becker B. (2024). Microbial response to seasonal variation in Arctic biocrusts with a focus on fungi and cyanobacteria. Environ. Res..

[20] Xu X., Qiu Y., Zhang K., Yang F., Chen M., Luo X., Yan X., Wang P., Zhang Y., Chen H. (2022). Climate warming promotes deterministic assembly of arbuscular mycorrhizal fungal communities. Glob. Change Biol..

[21] Lai Y., Zheng W., Zheng Y., Lu H., Qu S., Wang L., Li M., Wang S. (2024). Unveiling a novel entry gate: Insect foregut as an alternative infection route for fungal entomopathogens. Innovation.

[22] Chen J., Li W., Wu C., Wu S., Tong Y. (2025). Analysis of the Effects of *Beauveria bassiana* Appressorium formation on insect cuticle metabolism based on LC-MS. J. Fungi.

[23] Meng Y., Don P.D.W.H., Wang D. (2025). Isolation of 8 strains of *Beauveria* and the activity of their polysaccharides antioxidation and protective activity against H2O2-induced oxidative damage. Int. J. Biol. Macromol..

[24] Rahbek C. (1995). The elevational gradient of species richness: A uniform pattern?. Ecography.

[25] Mateus P., Sousa F., Martins M., Sousa B., Afonso A., Oliveira F., Moutinho-Pereira J., Fidalgo F., Soares C. (2024). The ectomycorrhizal fungus *Paxillus involutus* positively modulates *Castanea sativa* Miller (var. Marsol) responses to heat and drought co-exposure. Plant Physiol. Biochem..

[26] Xie L., Yang Y., Ma J., Lin G., Deng J., Robson T.M., Peng H., Zhou L., Yu D., Wang Q.W. (2024). Variations in ectomycorrhizal exploration types parallel seedling fine root traits of two temperate tree species under extreme drought and contrasting solar radiation treatments. Plant Cell Environ..

[27] Gao J.-L., Ge Y.-P., Matheny P.B., He P.-M., Wu X.-P., Bau T., Yu W.-J., Fan Y.-G. (2025). A phylogeny of the *Inocybe alienospora* group (Agaricales) with emphasis on seven new species from China and emendation of sect. Leptocybe. Mycology.

[28] Gao J.-L., Wu X.-P., Zhou Y.-L., Yu W.-J., Fan Y.-G. (2025). Additions to the *Inocybe* sect. *Leptocybe* (Agaricales) in China: New species from tropical rainforests, new geographical distributions, and toxin detection. Front. Microbiol..

[29] Schäfer T., Haun F., Rupp B., Hoffmeister D. (2025). Dissimilar reactions and enzymes for psilocybin biosynthesis in *Inocybe* and *Psilocybe* mushrooms. Angew. Chem. Int. Ed..

[30] Luo Y., Sheng X., Wei D., Song S., Chen C., Wu H., Shang J., Wang C. (2025). A binary-distributed effector modulates fungal host preference for drosophilids by targeting a lineage-specific immune factor. Proc. Natl. Acad. Sci. USA.

[31] Huang W., Huang P., Yü D., Li C., Huang S., Qi P., Huang S., Keyhani N.O., Huang Z. (2022). Proteomic analysis of a hypervirulent mutant of the insect-pathogenic fungus *Metarhizium anisopliae* reveals changes in pathogenicity and terpenoid pathways. Microbiol. Spectr..

[32] Zhang X., Meng Y., Huang Y., Zhang D., Fang W. (2021). A novel cascade allows *Metarhizium robertsii* to distinguish cuticle and hemocoel microenvironments during infection of insects. PLoS Biol..

[33] Faust K., Raes J. (2012). Microbial interactions: From networks to models. Nat. Rev. Microbiol..

[34] Wagg C., Schlaeppi K., Banerjee S., Kuramae E.E., van der Heijden M.G. (2019). Fungal-bacterial diversity and microbiome complexity predict ecosystem functioning. Nat. Commun..

[35] Yuan M.M., Guo X., Wu L., Zhang Y., Xiao N., Ning D., Shi Z., Zhou X., Wu L., Yang Y. (2021). Climate warming enhances microbial network complexity and stability. Nat. Clim. Change.

[36] Banerjee S., Schlaeppi K., Van Der Heijden M.G. (2018). Keystone taxa as drivers of microbiome structure and functioning. Nat. Rev. Microbiol..

